# Correlates of Total and domain-specific Sedentary behavior: a cross-sectional study in Dutch adults

**DOI:** 10.1186/s12889-020-8316-6

**Published:** 2020-02-12

**Authors:** Esmée A. Bakker, Maria T. E. Hopman, Duck-chul Lee, André L. M. Verbeek, Dick H. J. Thijssen, Thijs M. H. Eijsvogels

**Affiliations:** 1 Department of Physiology, Radboud Institute for Health Sciences, Radboud University Medical Center, P.O. Box 9101, 6500 HB Nijmegen, The Netherlands; 2 Research Institute for Sports and Exercise Sciences, Liverpool John Moores University, Liverpool, UK; 3 Department of Kinesiology, Iowa State University, Ames, USA; 4 Department for Health Evidence, Radboud Institute for Health Sciences, Radboud University Medical Center, Nijmegen, The Netherlands

**Keywords:** Sedentary behavior, Sitting time, Physical activity, Self-report, Cardiovascular diseases, Healthy lifestyle

## Abstract

**Background:**

Sedentary behavior (SB) is associated with increased risks of detrimental health outcomes. Few studies have explored correlates of SB in physically active individuals. Furthermore, SB correlates may depend on settings of SB, such as occupation, transportation and leisure time sitting. This study aims to identify subject-, lifestyle- and health-related correlates for total SB and different SB domains: transportation, occupation, and leisure time.

**Methods:**

Dutch participants were recruited between June, 2015 and December, 2016. Participant characteristics (i.e. age, sex, weight, height, marital status, education level, employment), lifestyle (sleep, smoking, alcohol consumption, physical activity) and medical history were collected via an online questionnaire. SB was assessed using the Sedentary Behavior Questionnaire and estimated for 9 different activities during weekdays and weekend days. Logistic regression was used to calculate odds ratios and 95% confidence intervals for the association between correlates and SB. Total SB was dichotomized at > 8 h/day and > 10 h/day, and being sedentary during transportation, occupation and leisure time at the 75th percentile (60 min/day, 275 min/day and 410 min/day, respectively).

**Results:**

In total, 8471 participants (median age 55, 55% men) were included of whom 86% met the physical activity guidelines. Median SB was 9.1 h/day (Q_25_ 6.3-Q_75_ 12.0) during weekdays and 7.4 h/day (Q_25_ 5.5-Q_75_ 9.5) during weekend days. SB was most prevalent during leisure time (5.3 h/day; Q_25_ 3.9-Q_75_ 6.8), followed by occupation (2 h/day; Q_25_ 0.1-Q_75_ 4.6) and transportation (0.5 h/day; Q_25_ 0.2-Q_75_ 1.0). Younger age, male sex, being unmarried, higher education, employment and higher BMI were significantly related to higher levels of total SB. Younger age, male sex, employment, and higher BMI increased the odds for high SB volumes during occupation and transportation. Higher education, being unmarried and smoking status were positively associated with high volumes of occupational SB only, whereas older age, being unmarried, unemployment, higher BMI and poor health were positively linked to leisure time SB.

**Conclusions:**

SB is highly prevalent in physically active individuals, with SB during leisure time as the most important contributor. Correlates for high volumes of SB vary substantially across SB domains, emphasizing the difficulty to target this unhealthy lifestyle.

## Background

Physical inactivity importantly contributes to the development of non-communicable diseases, such as cardiovascular diseases (CVD), type 2 diabetes, and breast and colon cancer [1]. In addition, evidence for deleterious health effects of sedentary behavior is rapidly accumulating. SB includes any waking behavior characterized by an energy expenditure ≤1.5 METs while in a sitting or reclining posture [2–4]. For example, sedentariness is associated with increased risks for all-cause mortality and the incidence of CVD, cancer, and type 2 diabetes [5, 6]. These observations emphasize the importance of SB as a highly prevalent, independent and modifiable risk factor for all-cause mortality and non-communicable diseases.

To enable effective reductions in SB via interventions or public health campaigns [7], identification of SB correlates is needed. A recent systemic review suggested that age, body mass index (BMI), physical activity levels, mood and attitude were associated with sedentariness [8]. However, identification of these correlates is primarily based on the general population, largely consisting of individuals not meeting the recommended physical activity guidelines. Previous work found physical activity could attenuate the adverse effects of sedentary behavior, but this was only present in individuals performing > 35.5 MET-hours/week (60–75 min of moderate intensity activity per day) [9]. Therefore, higher levels of sedentary behavior may have deleterious health effects in physically active individuals performing < 35.5 MET-hours/week. Correlates related to sedentary behavior may differ between physically inactive versus active individuals. Furthermore, SB correlates may depend on settings of SB, such as occupation, transportation and leisure time sitting [8, 10]. Better understanding of correlates of sedentary time, but also its dependency on the specific domains of SB, is needed to develop and implement interventions targeting SB.

We explored correlates of sedentary time in relation to the different settings of occupation, transportation, leisure time in a population with a wide range of physical activity levels. We hypothesized that subject-, lifestyle- and health-related correlates, such as identified in previous studies in the general population, also relate to SB in a physically active population. However, we expected that the presence and magnitude of these associations would be different across SB domains.

## Methods

### Study population

The Nijmegen Exercise Study is based on a cohort of individuals participating in Dutch sport events (i.e. International Nijmegen Four Days Marches and the Seven Hills Run) and their family and friends. The Nijmegen Exercise Study aims to investigate the impact of physical activity on health. Online questionnaires were used to inquire participants about demographic characteristics, anthropometric measures, lifestyle factors, and health status. All Dutch-speaking adults were eligible for the study. Participants were recruited via newsletters and internet advertisements between June 1, 2015 and December 31, 2016. A total number of 8952 participants completed the online questionnaire. After exclusion of participants with missing data for date of birth (*n* = 2) and sex (*n* = 1), or women who were pregnant (*n* = 45), 8904 participants remained available for inclusion. Another 433 participants were excluded for insufficient completion of the SB questionnaire, which resulted in 8471 participants being eligible for statistical analyses. The study (NL36743.091.11) was approved by The Local Committee on Research Involving Human Subjects of the region Arnhem and Nijmegen, the Netherlands. All participants provided written informed consent.

### Questionnaire

The online questionnaire asked participants about general characteristics, lifestyle factors and their medical history. General characteristics contained age, sex, weight, height, marital status, level of education and employment status. Lifestyle factors included sleeping hours, smoking behavior, alcohol consumption, and habitual physical activity. Smoking status was categorized into individuals who never smoked, smoked in the past (former smokers), and currently smoking. Heavy alcohol drinking was defined as > 14 alcoholic drinks per week for men, and > 7 for women [11]. Physical activity was measured with the SQUASH questionnaire [12]. Weekly physical activity was converted into METs and multiplied by minutes per week. MET minutes per week were classified into four categories: inactive (0 MET min/week), insufficient (1–499 MET min/week), medium (500–999 MET min/week), and high (≥1000 MET min/week) based on the 2018 US Physical Activity Guidelines [13]. In addition, participants were asked to subjectively describe their health status (very good, good, reasonable, fair, poor), and whether they had a physician confirmed diagnosis of CVD (myocardial infarction, stroke, or heart failure), cancer, or cardiovascular risk factors (hypertension, hypercholesterolemia, or diabetes mellitus).

### Assessment of sedentary behavior

Sedentary time was assessed using the Sedentary Behavior Questionnaire [14]. Sedentary time was estimated for nine different activities: watching television, playing computer/video games, sitting during eating and drinking, sitting while listening to music, sitting and talking on the phone, doing paperwork or office work, sitting and reading, sitting and playing a musical instrument or doing arts and crafts, sitting and driving/riding in a car, bus, or train. The nine items were completed for weekdays and weekend days separately, and stratified into three domains (occupation, transportation and leisure time). Sitting during occupation consisted of doing paperwork or office work; sedentary time during transportation contained sitting and driving/riding in a car, bus, or train; and leisure time sitting consisted of watching television, playing computer/video games and sitting during eating and drinking, sitting while listening to music, sitting and talking on the phone, sitting during reading, and sitting during playing a musical instrument or doing arts and crafts. Total sedentary behavior was based on the sum of the nine items per weekday and weekend day. The average amount of sedentary time per day was calculated by multiplying weekdays estimates by 5 and weekend days estimates by 2 and dividing this by 7. Since there are no thresholds for high engagement in sedentary time, we defined a high amount of sedentary time as > 8 h/day and > 10 h/day based on previous literature [15–17]. High amount of sedentary time during transportation, occupation and leisure time sitting were based on the 75% percentile (i.e. 1 h/day, 4 h and 25 min/day, and 6 h 50 min/day, respectively). Individuals, who were unemployed or retired, were excluded from the analyses regarding high levels of occupational sedentary time.

### Statistical analysis

Baseline characteristics were summarized as mean and SD or median and interquartile range for continuous variables, and as number and percentage for categorical variables. Associations with age, sex, marital status, education level, employment, BMI, smoking status, heavy alcohol drinking, physical activity and disease history were tested separately for total sedentary time and each setting of sedentary time using multivariable logistic regression analysis. We performed a complete case analysis (*n* = 7648), and analysis using multiple imputation (*n* = 8471) because 10% (*n* = 823) of the cases had missing values for one or more correlates. Missing data was imputed with multivariable imputation by chained equations with predictive mean matching. We checked patterns of missing data and followed the ‘missing at random’ assumption. All available variables were used to predict missing values in 10 imputed datasets with 100 burn-in iterations. Healthy convergence, imputed distribution and plausibility were verified. Furthermore, pooled estimates were derived from the 10 imputed datasets.

In addition, we analyzed the association of the previously mentioned correlates with continuous hours of total sedentary time and domains-specific sedentary time using multivariable linear regression analysis. Since sedentary time had a skewed distribution, we transformed sedentary time with the natural logarithm. Furthermore, we performed stratified analyses for active (MET min/week ≥500) and inactive (MET min/week < 500) individuals. All statistical tests were 2-sided, and significance was set at *P* < .05. All analyses were performed using SAS statistical software, version 9.4 (SAS Institute).

## Results

### Study population

The study population had a median age of 55 years (Q_25_ 45, Q_75_ 64) and 55% were men (Table 1). The majority of the participants were married (79%), received higher or academic education (62%) and were employed (75%). Two thirds of the participants had normal weight (66%), more than half never smoked (54%). As intended for the purpose of this research, 86% of the participants met the 2018 US Physical Activity Guidelines [13]. The majority of the study population (90%) classified their health status as good or very good. In total, 7% of the participants had a history of CVD, 16% hypertension, 13% hypercholesterolemia, 4% diabetes mellitus, and 8% cancer.

**Table 1:** General Characteristics of the 8471 Participants

Characteristic		Number of missing data
*Subject*		
Age	55 (45–64)	0
Sex (male)	4629 (55%)	0
Marital status (married or registered partnership)	6652 (79%)	43
Education		58
Low	687 (8%)	
Intermediate	2526 (30%)	
High/academic	5200 (62%)	
Employment (yes)	6320 (75%)	43
BMI (kg/m^2^)		45
Normal weight (< 25 kg/m^2^)	5594 (66%)	
Overweight (25–29 kg/m^2^)	2432 (29%)	
Obesity (≥ 30 kg/m^2^)	400 (5%)	
*Lifestyle*		
Smoking status		48
Never smoker	4510 (54%)	
Previous smoker	3436 (41%)	
Current smoker	477 (6%)	
Heavy alcohol drinking (yes)	1544 (19%)	154
Sleeping hours per day	7.0 (6.5–8.0)	358
Physical activity guidelines		0
0 MET-min/week	3 (0%)	
1–499 MET-min/week	1162 (14%)	
500–999 MET-min/week	242 (3%)	
≥ 1000 MET-min/week	7064 (83%)	
Health status		54
Very good	1830 (22%)	
Good	5724 (68%)	
Fair	748 (9%)	
Moderate	94 (1%)	
Bad	21 (0%)	
*Disease history*		
Cardiovascular diseases	512	266
Myocardial infarction	202 (2%)	144
Heart failure	192 (2%)	180
Stroke	170 (2%)	170
Hypertension	1357 (16%)	147
Hypercholesterolemia	1061 (13%)	166
Diabetes Mellitus	301 (4%)	240
Cancer	664 (8%)	122
Lung	11	
Breast	115	
Intestinal	61	
Prostate	90	
Other	412	

Data was presented as median (Q_25_-Q_75_) or as number (%)

### Prevalence of sedentary behavior

**Fig. 1:**
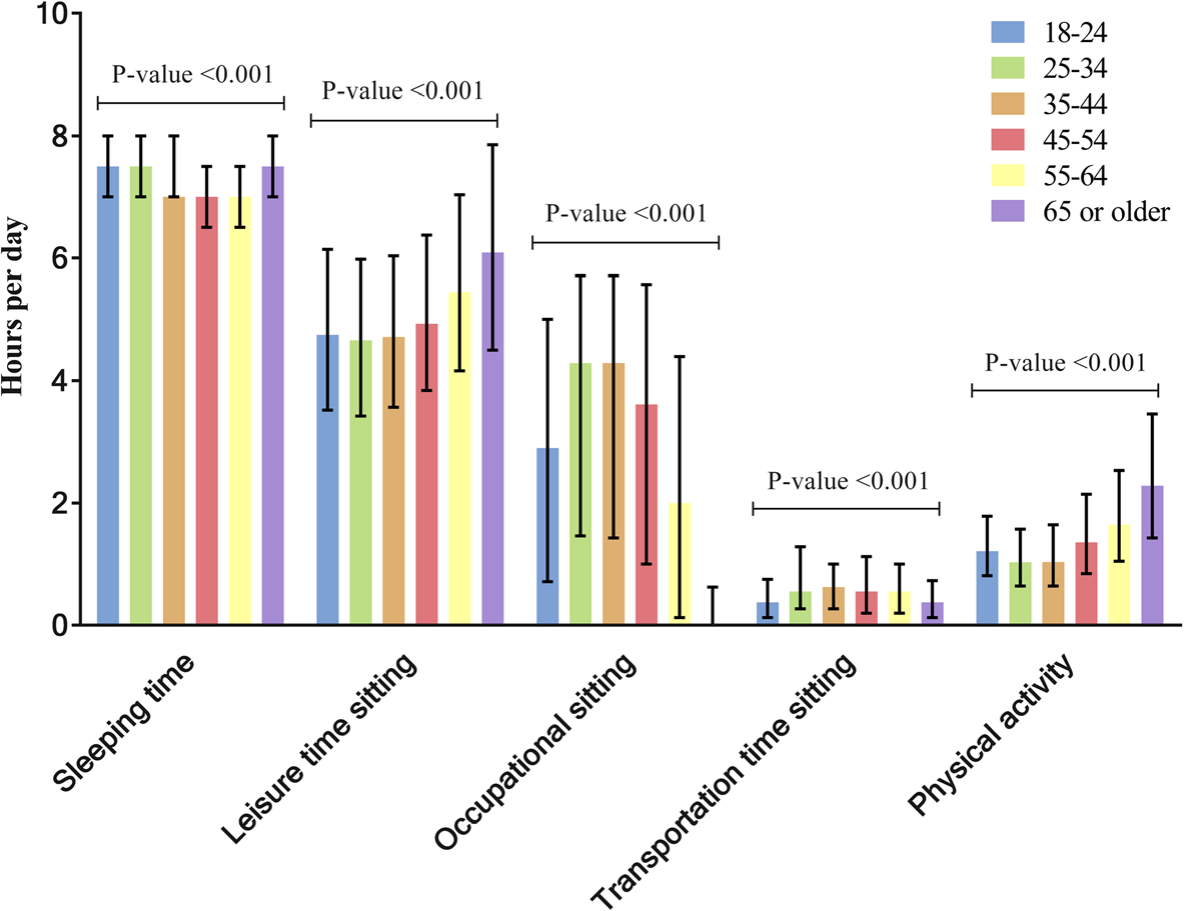
Time spent on sleeping, leisure time sitting, occupational sitting, transportation time sitting, and physical activity for different age categories (*N* = 8471). The median time spent on different activities is presented by bars and interquartile ranges by lines. Sedentary time during leisure time, occupation, and transportation, physical activity and sleeping time for were significantly different for the age categories (*P*-value Kruskal-Wallis < 0.001)

Participants reported a median sedentary time of 9.1 h (Q_25_ 6.3, Q_75_ 12.0) during weekdays and a median of 7.4 h (Q_25_ 5.5, Q_75_ 9.5) during weekend days. Sedentary time significantly differed across settings between weekdays and weekend days (*P* < .05). Median sedentary time at work was 2 h/day (Q_25_ 0.1, Q_75_ 6.0) on weekdays and 0 h/day (Q_25_ 0, Q_75_ 0.4) on weekend days. Median sedentary time during transportation was 0.4 h/day (Q_25_ 0.1, Q_75_ 1.0) on both weekdays and weekend days. Sedentary time during leisure time was 4.8 h/day (Q_25_ 3.5, Q_75_ 6.4) on weekdays and 6.25 h/day (Q_25_ 4.6, Q_75_ 8.1) on weekend days. The prevalence of domain specific sedentary time, physical activity and sleeping time differed significantly between age categories (*p*-value Kruskal-Wallis < 0.001, Fig. 1).

### Correlates of sedentary behavior

**Fig. 2:**
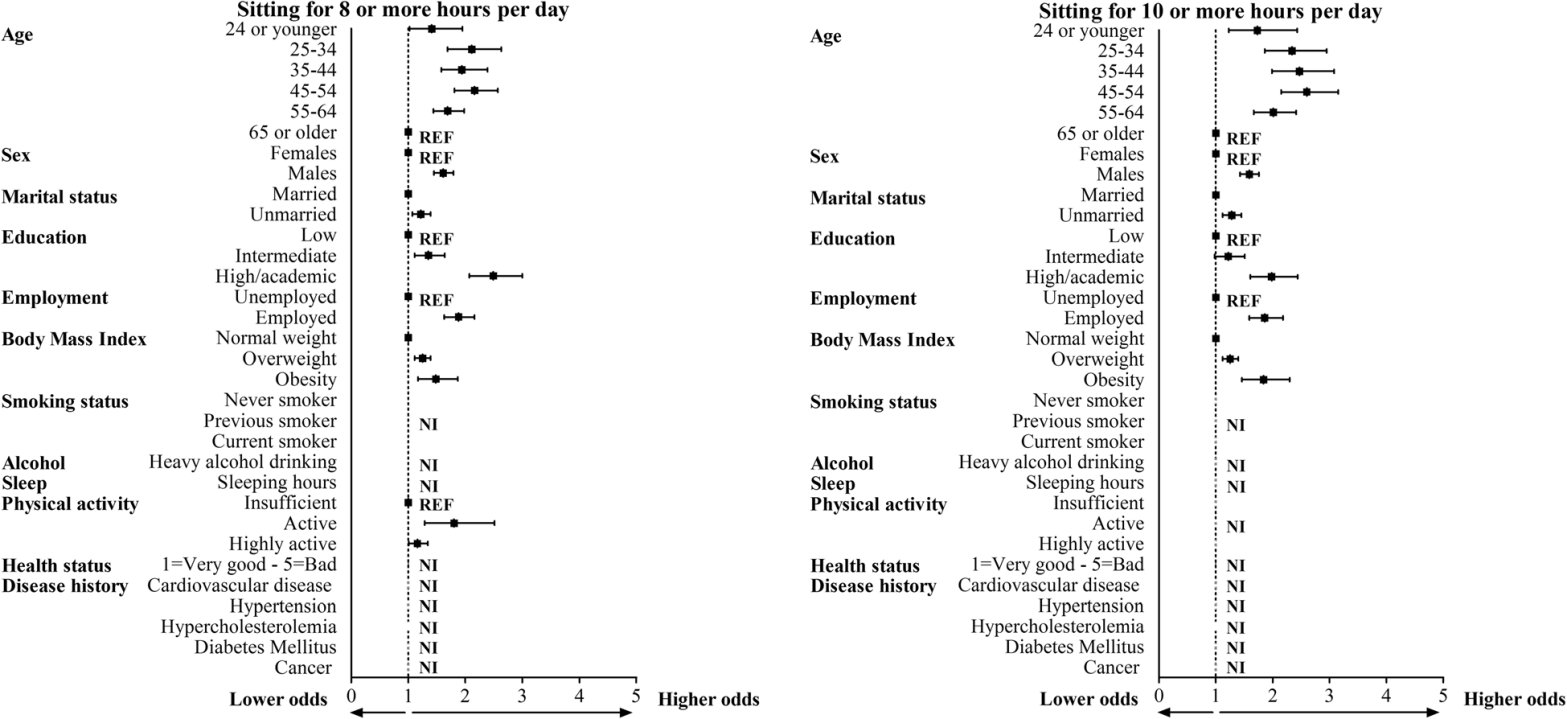
Correlates of sitting ≥8 or ≥ 10 h per day in the multivariate logistic regression models. The black squares indicate odds ratios and the lines 95% confidence intervals. All models included correlates which were significantly associated with sitting ≥8 or ≥ 10 h per day in the multivariate model. Correlates associated the sedentary time ≥ 8 or ≥ 10 h per day were comparable. NI = Not included in the multivariate regression model, REF = reference category

**Fig. 3:**
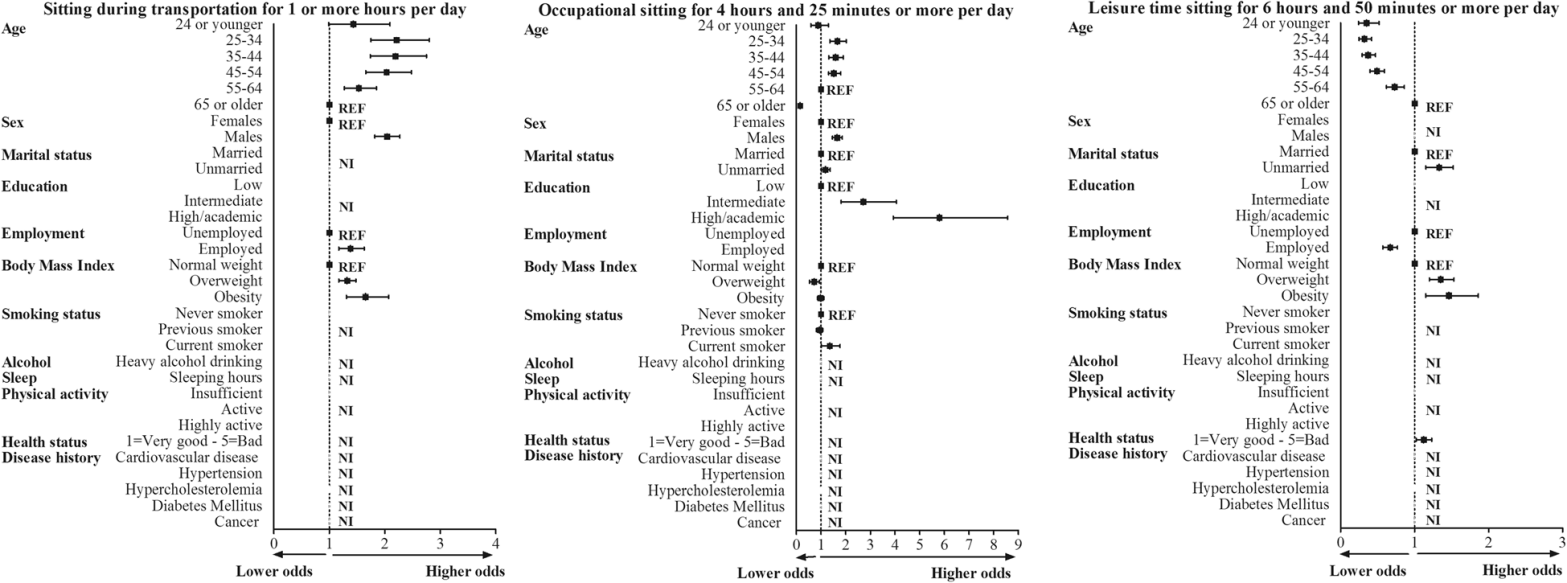
Correlates of sitting during transportation, occupation and leisure time in the multivariate model. The black squares indicate odds ratios and the lines 95% confidence intervals. All models included correlates which were significantly associated with sitting during transportation, occupation and leisure time in the multivariate model. Correlates associated the domain specific sedentary time differed within domains. NI = Not included in the multivariate regression model, REF = reference category

Younger age was significantly associated with higher odds of total sedentary time for ≥8 and ≥ 10 h/day, sedentary time during transportation and sedentary time at work (Figs. 2 and 3, and Table 2). This association reversed for sedentary time during leisure time, where we found that older age was associated with higher levels of sedentary time. Male sex was positively associated for all types of sedentary time, except for sedentary time during leisure time. Being unmarried was associated with higher levels of total amount of sedentary time and during transportation and occupation. Higher education was positively associated with total sedentary time and sedentary time at work, but not during transportation and leisure time.

**Table 2:** Estimates and 95% Confidence Intervals of the Multivariable Logistic Regression Analysis (Complete Case, N = 7648) for the Correlates of Sedentary Behaviour

	Total sedentary time ≥ 8 h per day			Total sedentary time ≥ 10 h per day			Transportation sedentary time ≥ 60 min			Occupational sedentary time ≥ 275 min*			Leisure sedentary time ≥ 410 min		
Characteristic	OR	Lower 95% CI	Upper 95% CI	OR	Lower 95% CI	Upper 95% CI	OR	Lower 95% CI	Upper 95% CI	OR	Lower 95% CI	Upper 95% CI	OR	Lower 95% CI	Upper 95% CI
*Subject*															
Age															
≤ 24	1.41	1.02	1.95	1.73	1.23	2.43	1.43	0.99	2.09	0.88	0.59	1.30	0.35	0.24	0.52
25–34	2.11	1.69	2.63	2.34	1.86	2.95	2.21	1.75	2.80	1.66	1.36	2.03	0.32	0.25	0.42
35–44	1.94	1.58	2.39	2.47	1.99	3.08	2.19	1.74	2.75	1.59	1.32	1.90	0.37	0.29	0.47
45–54	2.16	1.81	2.57	2.60	2.15	3.15	2.03	1.66	2.48	1.52	1.31	1.80	0.49	0.40	0.59
55–64	1.69	1.44	1.98	2.01	1.67	2.41	1.53	1.27	1.85	REF			0.73	0.62	0.86
≥ 65	REF			REF			REF			0.15	0.09	0.24	REF		
Sex (male)	1.61	1.45	1.79	1.59	1.43	1.76	2.04	1.82	2.27	1.65	1.46	1.86	1.33	1.15	1.52
Marital status (unmarried)	1.22	1.07	1.39	1.28	1.12	1.45	1.38	1.17	1.63	1.18	1.02	1.37	0.67	0.57	0.77
Education															
Low	REF			REF						REF					
Intermediate	1.35	1.11	1.64	1.22	0.98	1.51				2.71	1.82	4.05			
High/academic	2.49	2.07	3.00	1.98	1.61	2.44				5.80	3.93	8.57			
Employment (yes)	1.88	1.63	2.16	1.86	1.59	2.18									
BMI															
Normal weight	REF						REF			REF			REF		
Overweight	1.25	1.11	1.39		1.12	1.40	1.32	1.17	1.48	0.94	0.81	1.07	1.35	1.20	1.53
Obesity	1.48	1.17	1.87		1.46	2.30	1.65	1.31	2.07	1.35	1.02	1.77	1.46	1.15	1.86
*Lifestyle*															
Smoking status															
Never smoker										REF					
Previous smoker										0.72	0.54	0.94			
Current smoker										0.98	0.86	1.12			
Heavy alcohol drinking (yes)															
Sleeping hours per day															
Physical activity guidelines															
< 500 MET-min/week	REF														
500–999 MET-min/week	1.80	1.29	2.51												
≥ 1000 MET-min/week	1.16	1.01	1.34												
Health status (1 = very good – 5 = poor)													1.12	1.02	1.23
*Disease history*															
Cardiovascular diseases															
Hypertension															
Hypercholesterolemia															
Diabetes Mellitus															
Cancer										0.75	0.56	0.99			

All models included correlates which were significantly associated with sedentary time or domain-specific sedentary time in the multivariable model

Individuals who were unemployed and retired were excluded from this analysis (*N* = 6320)

BMI showed a clear positive association with higher levels of sedentary time for all domains, where the odds increased with higher BMI categories. Physical activity and smoking status were the only lifestyle factors that were significantly associated with sedentary time. Higher levels of physical activity increased the odds of total sedentary time for ≥8 h/day; being a former smoker decreased the odds for sitting at work. CVD and CVD risk factors were not associated with sedentary time. On the other hand, cancer was negatively associated with higher levels of occupational sedentary time. Poor health status was associated with higher odds of sedentary time during leisure time.

Results of the imputation analyses were almost similar compared to the complete case analyses. Except for former smoking status and cancer, which were both associated with higher odds of leisure time sitting (Additional file 1: Table S1). In addition, BMI was borderline statistically significant with occupational sedentary time. Results of linear regression analyses and the stratified analyses (physically active versus inactive individuals) largely confirmed our main analyses (Additional file 1: Table S2 and S3).

## Discussion

In our cohort we observed a number of new findings. First, a high level of SB was reported (9.1 h/day at weekdays; 7.4 h/day at weekend days), despite 86% of our population meeting the recommended physical activity dose. Second, we found that the majority of sedentary time was spent during leisure time activities and not at work. Third, younger age, male sex, being unmarried, higher education level, being employed, a higher BMI and higher physical activity levels were independently associated with higher levels of total SB, although this link was not consistently present across all SB domains. Other factors such as smoking status, cancer and health status were associated with specific domains of SB. Our results suggest that, SB is highly prevalent in physically active subjects and that correlates of SB differ across the various domains. Interventions to reduce SB might benefit from domain-specific targets, whilst the relative importance of these SB domains may differ between groups and/or individuals.

### Prevalence of sedentary behavior

In our study, we found a median total sedentary time of 9.1 h/day on weekdays and 7.4 h/day on weekend days. These results highlight that SB is not only present in the general population, but also highly prevalent in physically active individuals. This observation suggests that public health interventions to reduce sedentary behavior should be developed on a population-wide scale. Interestingly, the amount of sedentary time in our physically active cohort was ~ 1–4 h/day higher compared to previous studies [10, 18–21]. This discrepant finding may relate to the observation that Dutch individuals sit more compared to other European countries [18].

Interestingly, most sedentary time was spent during leisure time activities rather than during work. This observation differs from previous findings [21–23]. The difference might be partially explained by the relatively high percentage of older individuals who stopped working (25%) in our population compared to previous articles [21–23] (all working adults). Another explanation relates to differences in the physical activity levels of the profession, but most individuals in our population were highly educated, who typically perform desk-based office work. Alternatively, occupational sitting time was derived from a single item in the questionnaire, whereas leisure time sitting was calculated from seven items. Hence, study participants may have been reluctant to score a high sedentary time on a single item. Another study using the Sedentary Behavior Questionnaire also found higher levels of leisure time sitting compared to sedentary time at work [14]. Nonetheless, our observations may have important implications for SB interventions in physically active individuals. Since the time spent sedentary during leisure time is significantly higher compared to occupational sitting, workplace interventions for reducing total sedentary time might have limited effects in our population. Possibly, interventions focused on reducing SB during leisure time (e.g. watching TV, eating and drinking, and computer use) may be more relevant, especially since this type of SB counts for 51% of the total SB time in our population.

### Correlates of total and domain-specific sedentary behavior

Younger age, male sex, being unmarried, higher education level, being employed and a higher BMI were independently associated with higher levels of total SB. SB correlates identified in the present study align with previous findings [8, 21–26], but the direction of the association is different for age, smoking status and physical activity. In our study, age was negatively associated with high levels of sedentary time, which is in contrast to other studies, which found a positive association [20, 27]. A potential explanation for this distinction is that adults in our study, aged 25–64, reported much higher levels of sedentary time (median 9.3 h/day), compared to other studies (4–8 h/day) [18, 20, 27]. In contrast, our older adult population reported relatively lower levels of sedentary time [28]. A previous study found that retired individuals had higher levels of leisure time SB and physical activity [29], which is comparable with our study results in individuals of ≥65 years old. Former smoking was associated with lower levels of occupational sedentary time and with higher levels of leisure time sitting, compared to individuals who never smoked. In addition, we did not find any association in smokers, which is surprising and may relate to the low number (*n* = 477, 6%) of smokers in our population. Previous literature has shown that current smoking was associated with TV viewing, but not with total sedentary time [30]. Finally, physical activity was only associated with total sedentary time and not in different domains of SB. A cross-sectional study in 34,555 working adults found that physically active individuals were less sedentary in all domains [23]. The difference between our study and previous findings might be explained by the fact that our participants are mostly active, so maybe the amount of physical activity does not influence domains of SB in an already active population. Another explanation for the discrepant findings of the present study may relate to differences in cohort characteristics. Our cohort includes individuals who received mostly higher education, reporting high levels of physical activity, a low smoking status (6%), and a high self-reported health status (90% good to very good), which is different from most general population cohorts [22–24, 31].

Poor health status related to higher volumes of leisure time sitting, which is similar with findings from other studies [31]. However, heath-related issues such as cardiovascular diseases, hypertension, hypercholesterolemia and diabetes mellitus were not associated with sedentary time, which is in contrast to other studies suggesting that patients with cardiovascular diseases and diabetes mellitus are more sedentary compared to healthy controls [32, 33]. Opposite associations were also found for the relationship between cancer and sedentary time [34]. An important difference between our study and previous work is that the majority of the patient population in our study is physically active, might have a healthier lifestyle (only 6% smokers) and had a good self-reported health status (90% reported good to very good), which is not the case in most general patient populations. This may partially explain the different findings in our population. Still, future studies are needed to confirm our results. Especially studies in large populations investigating combinations of different correlates using multivariable regression models are necessary, because most current studies investigated only small groups of correlates. At least, our results suggest that presence of disease by definition is not automatically related to larger volumes of SB.

In this study, we hypothesized that subject-, lifestyle- and health- characteristics relate to SB, but that the magnitude of these associations may differ across SB domains. Indeed, education levels were associated with occupational SB but not with leisure and transportation SB. In addition, the association with age and cancer had a different direction for each domain. These findings indicate that some, but not all, SB correlates are domain specific, suggesting that tailored interventions may be needed to reduce SB across different domains and in specific target groups. Stratified analyses between active and inactive individuals confirmed our main analyses, but some differences were found. For example, discrepant results were found for marital status, BMI, some factors of disease history and lifestyle. Further, in this study we found opposite directions for association between age, current smoking status and physical activity, and high levels of SB compared to other previous mentioned literature. Although the directions were similar for active and inactive individuals in this study, the magnitude was larger in the active individuals. Further studies should determine, whether these differences in associations between active and inactive individuals do exist or whether this due to a lower sample size of inactive individuals.

### Strengths and limitations

The strengths of this study include a large cohort of mostly physically active individuals with a broad range of potential correlates. In addition, we used an extended questionnaire to inquire SB in three domains. We also asked the participants about their sleeping time and their physical activities to get a 24-h overview. However, limitations of our study include self-reported data on SB, physical activity and disease history, which all may cause measurement errors. In addition, occupation SB was measures with only one items, whereas leisure time SB was measures with seven items. Self-reported SB may be underestimated [35] and self-reported physical activity [36] overestimated compared to objectively measured SB and physical activity. However, due to the sample size of the study it was not feasible to use objective measures for SB and physical activity. In addition, objective measures of SB could not distinguish between different domains in which SB could take place. Future studies are needed to examine correlates of objectively measured sedentary time and patterns of SB, but in order to distinguish between domains, objective measures should be combined with subjective measures. Finally, we performed cross-sectional analyses of the associations between subject characteristics and sedentary time. Although the aim of the study was not to examine causation, it could be informative to investigate which correlates are associated with reductions in sedentary time using repeated measurements of SB. This may further improve interventions which aim to reduce sedentary time.

## Conclusion

Our results show that SB is highly prevalent in a physically active population. In addition, younger age, male sex, being unmarried, higher education level, employment and higher BMI are independently associated with higher levels of total SB. Moreover, these factors appear to follow a domain-specific pattern, with most factors showing different relations to SB between occupation- and transportation-domains versus leisure-time. Other factors such as physical activity, smoking status, cancer and health status are associated with specific domains of SB, or only with total sedentary time. These observations indicate that interventions to reduce sedentary time should incorporate correlates for domain-specific SB to enhance the effect size and specifically target the most important domains of SB.

## Supplementary information

**Additional file 1: Table S1.** Estimates and 95% Confidence Intervals of the Multivariable Logistic Regression Analysis (Imputation Analyses, *N* = 8471) for the Correlates of Sedentary Behaviour. **Table S2.** Estimates and 95% Confidence Intervals of the Multivariable Linear Regression Analysis (Complete Case, *N* = 7648) for the Correlates of Sedentary Behaviour. **Table S3.** Odds Ratios and 95% Confidence Intervals of the Multivariable Logistic Regression Analysis (Complete Analyses, *N* = 7648) for the Correlates of Sedentary Behaviour Stratified for Active and Inactive Individuals.

## Data Availability

The datasets used and/or analyzed during the current study are available from the corresponding author on reasonable request.

## Abbrevations

BMI: Body Mass Index
CVD: Cardiovascular disease
MET: Metabolic Equivalent of Task
SB: Sedentary Behavior
