# Supplementary material for: Correlates of Total and domain-specific Sedentary behavior: a cross-sectional study in Dutch adults
Source: BMC Public Health. 2020 Feb 12;20:220. doi: 10.1186/s12889-020-8316-6 (PMC7017477; doi:10.1186/s12889-020-8316-6)
Supplement: Supplementary file 1 — Additional file 1: Table S1. Estimates and 95% Confidence Intervals of the Multivariable Logistic Regression Analysis (Imputation Analyses, N = 8471) for the Correlates of Sedentary Behaviour. Table S2. Estimates and 95% Confidence Intervals of the Multivariable Linear Regression Analysis (Complete Case, N = 7648) for the Correlates of Sedentary Behaviour. Table S3. Odds Ratios and 95% Confidence Intervals of the Multivariable Logistic Regression Analysis (Complete Analyses, N = 7648) for the Correlates of Sedentary Behaviour Stratified for Active and Inactive Individuals. [file 12889_2020_8316_MOESM1_ESM.docx]

**Additional material**

| Table S1. Estimates and 95% Confidence Intervals of the Multivariable Logistic Regression Analysis (Imputation Analyses, N=8471) for the Correlates of Sedentary Behaviour. | | | | | | | | | | | | | | | | | |
| --- | --- | --- | --- | --- | --- | --- | --- | --- | --- | --- | --- | --- | --- | --- | --- | --- | --- |
|  | Total sedentary time ≥8 hours per day | | | Total sedentary time ≥10 hours per day | | | Transportation sedentary time ≥60 min | | | Occupational sedentary time ≥ 275 min* | | | Leisure sedentary time ≥410 min | | | | |
| Characteristic | OR | Lower 95% CI | Upper 95% CI | OR | Lower 95% CI | Upper 95% CI | OR | Lower 95% CI | Upper 95% CI | OR | Lower 95% CI | Upper 95% CI | OR | | Lower 95% CI | | Upper 95% CI |
| *Subject* |  |  |  |  |  |  |  |  |  |  |  |  |  |  | |  | |
| Age  ≤24  25-34  35-44  45-54  55-64  ≥65 | 1.53  2.19  2.04  2.23  1.77  REF | 1.12  1.78  1.67  1.90  1.52 | 2.08  2.70  2.48  2.63  2.05 | 1.90  2.41  2.60  2.72  2.07  REF | 1.38  1.94  2.11  2.28  1.75 | 2.64  2.99  3.20  3.26  2.45 | 1.36  2.21  2.12  2.00  1.53  REF | 0.95  1.77  1.71  1.66  1.29 | 1.96  2.76  2.63  2.41  1.82 | 0.97  1.78  1.66  1.55  REF  0.12 | 0.66  1.46  1.39  1.35  0.08 | 1.42  2.15  1.98  1.78  0.20 | 0.35  0.36  0.40  0.53  0.77  REF | 0.24  0.28  0.32  0.45  0.66 | | 0.52  0.46  0.51  0.63  0.90 | |
| Sex (male) | 1.60 | 1.45 | 1.76 | 1.58 | 1.43 | 1.74 | 2.11 | 1.90 | 2.34 | 1.68 | 1.49 | 1.88 |  |  | |  | |
| Marital status (unmarried) | 1.22 | 1.08 | 1.37 | 1.24 | 1.10 | 1.40 |  |  |  | 1.16 | 1.01 | 1.34 | 1.33 | 1.17 | | 1.52 | |
| Education  Low  Intermediate  High/academic | REF  1.40  2.55 | 1.17  2.13 | 1.69  3.04 | REF  1.28  2.06 | 1.04  1.68 | 1.57  2.52 |  |  |  | REF  2.74  5.72 | 1.86  3.94 | 4.02  8.32 |  |  | |  | |
| Employment (yes) | 1.84 | 1.61 | 2.10 | 1.82 | 1.57 | 2.11 | 1.41 | 1.21 | 1.65 | 3.61 | 2.83 | 4.60 | 0.66 | 0.57 | | 0.76 | |
| BMI  Normal weight  Overweight  Obesity | REF  1.27  1.47 | 1.14  1.18 | 1.42  1.83 | REF  1.27  1.80 | 1.14  1.45 | 1.41  2.23 | 1.31  1.58 | 1.17  1.27 | 1.46  1.97 |  |  |  | REF  1.35  1.50 | 1.20  1.20 | | 1.50  1.89 | |
| *Lifestyle* |  |  |  |  |  |  |  |  |  |  |  |  |  |  | |  | |
| Smoking status  Never smoker  Previous smoker  Current smoker |  |  |  |  |  |  |  |  |  | REF  0.73  0.98 | 0.56  0.86 | 0.95  1.10 | REF  1.27  1.08 | 1.02  0.96 | | 1.58  1.20 | |
| Heavy alcohol drinking (yes) |  |  |  |  |  |  |  |  |  |  |  |  |  |  | |  | |
| Sleeping hours per day |  |  |  |  |  |  |  |  |  |  |  |  |  |  | |  | |
| Physical activity guidelines  <500 MET-min/week  500-999 MET-min/week  ≥1000 MET-min/week | REF  1.69  1.15 | 1.23  1.00 | 2.32  1.31 |  |  |  |  |  |  |  |  |  |  |  | |  | |
| Health status  (1 = very good – 5 = poor) |  |  |  |  |  |  |  |  |  |  |  |  | 1.10 | 1.01 | | 1.20 | |
| *Disease history* |  |  |  |  |  |  |  |  |  |  |  |  |  |  | |  | |
| Cardiovascular diseases |  |  |  |  |  |  |  |  |  |  |  |  |  |  | |  | |
| Hypertension |  |  |  |  |  |  |  |  |  |  |  |  |  |  | |  | |
| Hypercholesterolemia |  |  |  |  |  |  |  |  |  |  |  |  |  |  | |  | |
| Diabetes Mellitus |  |  |  |  |  |  |  |  |  |  |  |  |  |  | |  | |
| Cancer |  |  |  |  |  |  |  |  |  |  |  |  | 1.20 | 1.01 | | 1.43 | |

All models included correlates which were significantly associated with sedentary time or domain-specific sedentary time in the multivariable model.

* Individuals who were unemployed and retired were excluded from this analysis (N= 6320)

Table S2. Estimates and 95% Confidence Intervals of the Multivariable Linear Regression Analysis (Complete Case, N=7648) for the Correlates of Sedentary Behaviour.

| LN transformation | Total sedentary hours per day | | | | Transportation sedentary time ≥60 min | | | | Occupational sedentary time ≥ 275 min* | | | | Leisure sedentary time ≥410 min | | | | | | | |
| --- | --- | --- | --- | --- | --- | --- | --- | --- | --- | --- | --- | --- | --- | --- | --- | --- | --- | --- | --- | --- |
| Characteristic | Beta | *e*^Beta^ | Lower 95% CI | Upper 95% CI | Beta | *e*^Beta^ | Lower 95% CI | Upper 95% CI | Beta | *e*^Beta^ | Lower 95% CI | Upper 95% CI | | Beta | | *e*^Beta^ | | Lower 95% CI | | Upper 95% CI |
| *Subject* |  |  |  |  |  |  |  |  |  |  |  |  |  | |  | |  | |  | |
| Age  ≤24  25-34  35-44  45-54  55-64  ≥65 | 0.06  0.14  0.15  0.16  0.12  REF | 1.07  1.15  1.17  1.18  1.13 | 0.00  0.10  0.11  0.13  0.09 | 0.13  0.18  0.20  0.20  0.16 | 0.13  0.36  0.36  0.31  0.18  REF | 1.14  1.44  1.44  1.36  1.20 | -0.03  0.26  0.26  0.22  0.10 | 0.29  0.47  0.46  0.40  0.26 | 0.01  0.18  0.16  0.15  REF  -0.62 | 1.20  1.17  1.16  1.00 | -0.17  0.09  0.08  0.08  0.07 | 0.19  0.28  0.25  0.22  0.22 | -0.22  -0.22  -0.18  -0.13  -0.06  REF | | 0.80  0.80  0.83  0.87  0.94 | | -0.29  -0.27  -0.23  -0.17  -0.10 | | -0.15  -0.18  -0.14  -0.10  -0.03 | |
| Sex (male) | 0.10 | 1.11 | 0.08 | 0.12 | 0.10 | 1.42 | 0.30 | 0.40 | 0.28 | 1.32 | 0.22 | 0.34 |  | |  | |  | |  | |
| Marital status (unmarried) | 0.05 | 1.06 | 0.03 | 0.08 | 0.35 | 1.10 | 0.04 | 0.16 |  |  |  |  | 0.05 | | 1.05 | | 0.02 | | 0.08 | |
| Education  Low  Intermediate  High/academic | REF  0.08  0.20 | 1.08  1.22 | 0.04  0.16 | 0.12  0.24 |  |  |  |  | REF  0.62  1.24 | 1.86  3.46 | 0.47  1.10 | 0.77  1.32 | REF 0.04  0.03 | | 1.04  1.03 | | 0.00  -0.01 | | 0.08  0.07 | |
| Employment (yes) | 0.12 | 1.13 | 0.09 | 0.15 | 0.18 | 1.20 | 0.11 | 0.25 |  |  |  |  | -0.07 | | 0.94 | | -0.10 | | -0.04 | |
| BMI  Normal weight  Overweight  Obesity | REF  0.03  0.09 | 1.04  1.10 | 0.01  0.05 | 0.06  0.14 | REF  0.13  0.28 | 1.14  1.32 | 0.08  0.16 | 0.19  0.39 |  |  |  |  | REF  0.04  0.08 | | 1.04  1.08 | | 0.02  0.03 | | 0.06  0.13 | |
| *Lifestyle* |  |  |  |  |  |  |  |  |  |  |  |  |  | |  | |  | |  | |
| Smoking status  Never smoker  Previous smoker  Current smoker |  |  |  |  |  |  |  |  |  |  |  |  |  | |  | |  | |  | |
| Heavy alcohol drinking (yes) |  |  |  |  |  |  |  |  |  |  |  |  | 0.02 | | 1.02 | | 0.00 | | 0.05 | |
| Sleeping hours per day |  |  |  |  |  |  |  |  |  |  |  |  |  | |  | |  | |  | |
| Physical activity guidelines  <500 MET-min/week  500-999 MET-min/week  ≥1000 MET-min/week | REF  0.07  0.01 | 1.07  1.01 | 0.01  -0.01 | 0.13  0.04 | REF  0.16  -0.06 | 1.17  0.95 | 0.01  -0.13 | 0.30  0.01 | REF  0.24  0.10 | 1.27  1.11 | 0.07  0.01 | 0.40  0.18 |  | |  | |  | |  | |
| Health status  (1 = very good – 5 = poor) |  |  |  |  |  |  |  |  |  |  |  |  |  | |  | |  | |  | |
| *Disease history* |  |  |  |  |  |  |  |  |  |  |  |  |  | |  | |  | |  | |
| Cardiovascular diseases |  |  |  |  |  |  |  |  |  |  |  |  |  | |  | |  | |  | |
| Hypertension |  |  |  |  |  |  |  |  |  |  |  |  |  | |  | |  | |  | |
| Hypercholesterolemia |  |  |  |  |  |  |  |  |  |  |  |  |  | |  | |  | |  | |
| Diabetes Mellitus | 0.06 | 1.06 | 0.01 | 0.11 |  |  |  |  | -0.05 | 0.95 | -0.10 | -0.01 | 0.07 | | 1.07 | | 0.01 | | 0.12 | |
| Cancer |  |  |  |  |  |  |  |  |  |  |  |  | 0.03 | | 1.03 | | 0.02 | | 0.05 | |

All models included correlates which were significantly associated with sedentary time or domain-specific sedentary time in the multivariable model.
* Individuals who were unemployed and retired were excluded from this analysis (N= 6320)

| Table S3. Odds Ratios and 95% Confidence Intervals of the Multivariable Logistic Regression Analysis (Complete Analyses, N=7648) for the Correlates of Sedentary Behaviour Stratified for Active and Inactive Individuals. | | | | | | | | | | | | | | | | | | |
| --- | --- | --- | --- | --- | --- | --- | --- | --- | --- | --- | --- | --- | --- | --- | --- | --- | --- | --- |
|  | Total sedentary time ≥8 hours per day | | | Total sedentary time ≥10 hours per day | | | Transportation sedentary time ≥60 min | | | Occupational sedentary time ≥ 275 min* | | | Leisure sedentary time ≥410 min | | | | | |
| Characteristic | OR | Lower 95% CI | Upper 95% CI | OR | Lower 95% CI | Upper 95% CI | OR | Lower 95% CI | Upper 95% CI | OR | Lower 95% CI | Upper 95% CI | | OR | | Lower 95% CI | | Upper 95% CI |
| *Active individuals* |  |  |  |  |  |  |  |  |  |  |  |  |  | |  | |  | |
| *Subject* |  |  |  |  |  |  |  |  |  |  |  |  |  | |  | |  | |
| Age  ≤24  25-34  35-44  45-54  55-64  ≥65 | 1.83  2.38  2.04  2.19  1.81  REF | 1.32  1.90  1.63  1.82  1.52 | 2.54  3.00  2.55  2.65  2.15 | 1.74  2.21  2.36  2.46  1.90  REF | 1.22  1.73  1.87  2.00  1.57 | 2.49  2.82  2.99  3.02  2.32 | 1.35  2.13  2.18  2.02  1.54  REF | 0.90  1.66  1.70  1.63  1.26 | 2.02  2.74  2.78  2.51  1.89 | 1.01  1.73  1.62  1.55  REF  0.17 | 0.67  1.40  1.33  1.33  0.11 | 1.52  2.13  1.96  1.81  0.27 | 0.32  0.33  0.36  0.47  0.72  REF | | 0.21  0.25  0.28  0.39  0.60 | | 0.49  0.43  0.46  0.58  0.86 | |
| Sex (male) | 1.58 | 1.42 | 1.76 | 1.57 | 1.41 | 1.75 | 2.01 | 1.78 | 2.26 | 1.62 | 1.42 | 1.83 |  | |  | |  | |
| Marital status (unmarried) |  |  |  | 1.26 | 1.10 | 1.44 |  |  |  | 1.19 | 1.02 | 1.39 | 1.34 | | 1.15 | | 1.55 | |
| Education  Low  Intermediate  High/academic | REF 1.39  2.50 | 1.12  2.02 | 1.72  3.08 | REF 1.27  1.97 | 0.99  1.55 | 1.63  2.50 |  |  |  | REF  2.38  4.74 | 1.56  3.14 | 3.64  7.16 |  | |  | |  | |
| Employment (yes) | 1.88 | 1.61 | 2.19 | 1.86 | 1.57 | 2.21 | 1.40 | 1.17 | 1.68 | 3.89 | 2.95 | 5.13 | 0.65 | | 0.55 | | 0.76 | |
| BMI  Normal weight  Overweight  Obesity | REF  1.22  1.44 | 1.08  1.11 | 1.38  1.87 | REF  1.24  1.94 | 1.09  1.51 | 1.39  2.50 | REF  1.31  1.78 | 1.16  1.38 | 1.48  2.29 |  |  |  | REF  1.27  1.39 | | 1.12  1.06 | | 1.45  1.83 | |
| *Lifestyle* |  |  |  |  |  |  |  |  |  |  |  |  |  | |  | |  | |
| Smoking status  Never smoker  Previous smoker  Current smoker |  |  |  |  |  |  |  |  |  | REF  0.71  1.01 | 0.52  0.89 | 0.97  1.16 |  | |  | |  | |
| Heavy alcohol drinking (yes) |  |  |  |  |  |  |  |  |  |  |  |  |  | |  | |  | |
| Sleeping hours per day |  |  |  |  |  |  |  |  |  |  |  |  |  | |  | |  | |
| Physical activity guidelines  <500 MET-min/week  500-999 MET-min/week  ≥1000 MET-min/week |  |  |  |  |  |  |  |  |  |  |  |  |  | |  | |  | |
| Health status  (1 = very good – 5 = poor) |  |  |  |  |  |  |  |  |  |  |  |  | 1.15 | | 1.04 | | 1.27 | |
| *Disease history* |  |  |  |  |  |  |  |  |  |  |  |  |  | |  | |  | |
| Cardiovascular diseases |  |  |  |  |  |  |  |  |  |  |  |  |  | |  | |  | |
| Hypertension |  |  |  |  |  |  |  |  |  |  |  |  |  | |  | |  | |
| Hypercholesterolemia |  |  |  |  |  |  |  |  |  |  |  |  |  | |  | |  | |
| Diabetes Mellitus |  |  |  |  |  |  |  |  |  |  |  |  |  | |  | |  | |
| Cancer |  |  |  |  |  |  |  |  |  | 0.76 | 0.56 | 1.02 |  | |  | |  | |
| *Inactive individuals* |  |  |  |  |  |  |  |  |  |  |  |  |  | |  | |  | |
| *Subject* |  |  |  |  |  |  |  |  |  |  |  |  |  | |  | |  | |
| Age  ≤24  25-34  35-44  45-54  55-64  ≥65 | 0.66  1.86  1.86  2.33  1.23  REF | 0.24  1.01  1.03  1.42  0.81 | 1.80  3.42  3.34  3.83  1.86 | 1.36  3.24  2.96  3.68  2.72  REF | 0.43  1.65  1.55  2.09  1.66 | 4.23  6.37  5.68  6.47  4.47 | 2.70  3.37  2.58  2.53  1.69  REF | 0.97  1.89  1.49  1.64  1.14 | 7.55  6.03  4.46  3.89  2.50 |  |  |  | 0.61  0.26  0.35  0.47  0.68  REF | | 0.21  0.13  0.19  0.31  0.48 | | 1.73  0.53  0.64  0.71  0.95 | |
| Sex (male) | 1.69 | 1.26 | 2.25 | 1.77 | 1.31 | 2.40 | 2.37 | 1.73 | 3.24 |  |  |  |  | |  | |  | |
| Marital status (unmarried) |  |  |  |  |  |  |  |  |  |  |  |  |  | |  | |  | |
| Education  Low  Intermediate  High/academic | REF 1.13  2.54 | 0.74  1.70 | 1.73  3.80 | REF  0.85  1.87 | 0.52  1.19 | 1.39  2.95 |  |  |  | REF  4.29  14.70 | 1.28  4.51 | 14.44  47.94 |  | |  | |  | |
| Employment (yes) | 1.84 | 1.27 | 2.67 | 1.82 | 1.20 | 2.77 |  |  |  |  |  |  |  | |  | |  | |
| BMI  Normal weight  Overweight  Obesity | REF  1.42  1.79 | 1.06  1.05 | 1.89  3.03 |  |  |  |  |  |  |  |  |  | REF  1.86  1.96 | | 1.38  1.16 | | 2.51  3.31 | |
| *Lifestyle* |  |  |  |  |  |  |  |  |  |  |  |  |  | |  | |  | |
| Smoking status  Never smoker  Previous smoker  Current smoker |  |  |  | REF  0.85  0.72 | 0.53  0.52 | 1.36  0.99 |  |  |  | REF  0.79  0.73 | 0.42  0.49 | 1.48  1.10 |  | |  | |  | |
| Heavy alcohol drinking (yes) |  |  |  |  |  |  |  |  |  | 0.38 | 0.23 | 0.65 |  | |  | |  | |
| Sleeping hours per day |  |  |  |  |  |  |  |  |  |  |  |  |  | |  | |  | |
| Physical activity guidelines  <500 MET-min/week  500-999 MET-min/week  ≥1000 MET-min/week |  |  |  |  |  |  |  |  |  |  |  |  |  | |  | |  | |
| Health status  (1 = very good – 5 = poor) |  |  |  |  |  |  |  |  |  |  |  |  |  | |  | |  | |
| *Disease history* |  |  |  |  |  |  |  |  |  |  |  |  |  | |  | |  | |
| Cardiovascular diseases |  |  |  |  |  |  |  |  |  |  |  |  |  | |  | |  | |
| Hypertension |  |  |  |  |  |  |  |  |  |  |  |  |  | |  | |  | |
| Hypercholesterolemia |  |  |  |  |  |  |  |  |  |  |  |  |  | |  | |  | |
| Diabetes Mellitus |  |  |  |  |  |  |  |  |  |  |  |  |  | |  | |  | |
| Cancer |  |  |  |  |  |  |  |  |  |  |  |  |  | |  | |  | |

All models included correlates which were significantly associated with sedentary time or domain-specific sedentary time in the multivariable model.

* Individuals who were unemployed and retired were excluded from this analyses (N= 6320)
